# Live imaging of paracrine signaling: Advances in visualization and tracking techniques

**DOI:** 10.1247/csf.24064

**Published:** 2025-01-22

**Authors:** Eriko Deguchi, Michiyuki Matsuda, Kenta Terai

**Affiliations:** 1 Department of Pathology and Biology of Diseases, Graduate School of Medicine, Kyoto University, Yoshida-Konoe-cho, Sakyo-ku, Kyoto 606-8501, Japan; 2 Laboratory of Cell Cycle Regulation, Graduate School of Biostudies, Kyoto University, Yoshida-Konoe-cho, Sakyo-ku, Kyoto 606-8501, Japan; 3 Integrated Graduate School of Medicine, Engineering, and Agricultural Sciences, University of Yamanashi, Shimokato, Chuo-shi, Yamanashi 409-3898, Japan; 4 Department of Histology, Graduate School of Medicine, Tokushima University, 3-18-15 Kuramoto-cho, Tokushima 770-8503, Japan

**Keywords:** paracrine signaling, live imaging, biosensors, optogenetics, chemogenetics

## Abstract

Live imaging techniques have revolutionized our understanding of paracrine signaling, a crucial form of cell-to-cell communication in biological processes. This review examines recent advances in visualizing and tracking paracrine factors through four key stages: secretion from producing cells, diffusion through extracellular space, binding to target cells, and activation of intracellular signaling within target cells. Paracrine factor secretion can be directly visualized by fluorescent protein tagging to ligand, or indirectly by visualizing the cleavage of the transmembrane pro-ligands or plasma membrane fusion of endosomes comprising the paracrine factors. Diffusion of paracrine factors has been studied using techniques such as fluorescence correlation spectroscopy (FCS), fluorescence recovery after photobleaching (FRAP), fluorescence decay after photoactivation (FDAP), and single-molecule tracking. Binding of paracrine factors to target cells has been visualized through various biosensors, including GPCR-activation-based (GRAB) sensors and Förster resonance energy transfer (FRET) probes for receptor tyrosine kinases. Finally, activation of intracellular signaling is monitored within the target cells by biosensors for second messengers, transcription factors, and so on. In addition to the imaging tools, the review also highlights emerging optogenetic and chemogenetic tools for triggering the release of paracrine factors, which is essential for associating the paracrine factor secretion to biological outcomes during the bioimaging of paracrine factor signaling.

## Graphical Abstract

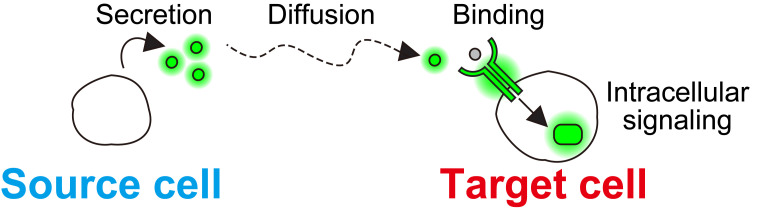

## Fundamentals and Classification of Paracrine Signaling

Understanding paracrine signaling is crucial in many areas of biology and medicine, considering its key roles in embryonic development, tissue homeostasis, and various pathological conditions including cancer progression and inflammatory responses. Despite its importance, studying paracrine signaling in physiological contexts presents unique challenges due to the difficulties in detecting paracrine factor secretion, tracking the small number of ligands released into the extracellular space, and associating the biological response with the released paracrine factors. Addressing these challenges requires advanced visualization techniques, which will be the focus of the following sections.

Intercellular communication mediated by signaling molecules secreted from source cells into the extracellular space is classified into at least three categories: synaptic signaling, paracrine signaling, and endocrine signaling. Endocrine signaling differs from paracrine signaling in that the signaling molecules in this class reach target cells through the bloodstream, allowing them to control distant organs. Synaptic signaling is excluded from paracrine signaling due to the specific structure and cell types (i.e., neurons) involved in this signaling mechanism. Because imaging of synaptic signaling has been reviewed elsewhere (Da *et al.*, 2023; Day-Cooney *et al.*, 2023; Muir *et al.*, 2024), we will primarily focus on paracrine signaling rather than synaptic signaling.

A specific form of paracrine signaling is autocrine signaling. In a strict definition, autocrine signaling occurs when a signaling molecule released from a cell binds to receptors expressed on/in the same cell. However, in many circumstances, it is difficult to distinguish whether the signaling molecules bound to a cell come from itself or neighboring cells of the same type; therefore, the broad definition of autocrine signaling includes paracrine signaling between cells of the same type. Notably, some paracrine signaling molecules can transmit signals to contacting cells while still on the membrane of the source cells, a process known as juxtacrine signaling. Thus, some phenomena ascribed to paracrine signaling may be caused by juxtacrine signaling.

**Fig. 1:**
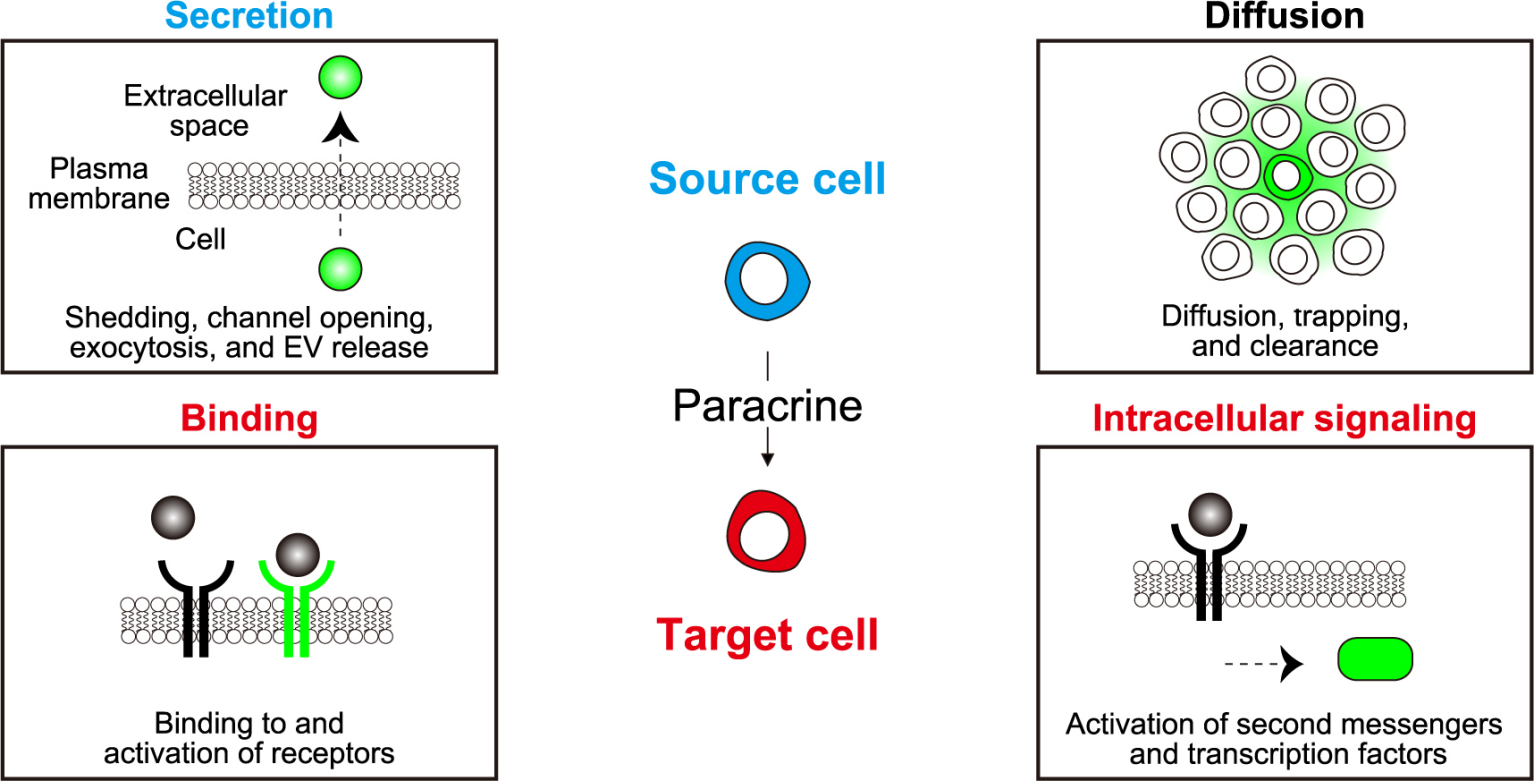
Visualization of the four key steps in paracrine signaling Secretion: Release of paracrine factors from source cells into the extracellular space. EV, extracellular vesicle. Diffusion: Spread of paracrine factors across multiple cells or throughout the tissue. Binding: Detection of paracrine factors by specific receptors on target cells. Intracellular signaling: Initiation of intracellular signaling cascades in response to paracrine factors.

Paracrine factors can be classified by various aspects: chemical nature, biological function, range of action, mechanism of action, etc. Among these, recent advances in fluorescence microscopy are opening a new window into understanding the range of action of paracrine signaling molecules. We will describe the methodology to visualize paracrine signaling in the following four categories (Fig. 1). The first step of paracrine signaling is secretion from the source cells. The signaling molecules may be proteins/peptides, lipids, or gases. These can be released from the source cells by shedding via proteases, secretion through channel opening, exocytosis of intracellular vesicles, free diffusion, or via exosomes. The second step is diffusion to reach the target cells. The third step is the binding to the cells. Notably, the binding to target cells does not guarantee successful information transmission, which can be visualized either by the activation of receptors or intracellular signaling pathways. We will provide an overview of the visualizing tools for these four steps.

## Techniques for Live Visualization of Paracrine Factor Secretion

First, we need to consider the labeling techniques for paracrine factors generated by the source cells of interest. For visualization under fluorescent microscopes, among the three categories of paracrine factors (peptides, lipids, and gases), only peptide ligands can be specifically labeled by recombinant DNA technology or chemical biology. A detailed description of labeling technologies is outside the scope of this review; therefore, in most cases, we will present fluorescent protein (FP)-tagged ligands as examples.

**Fig. 2:**
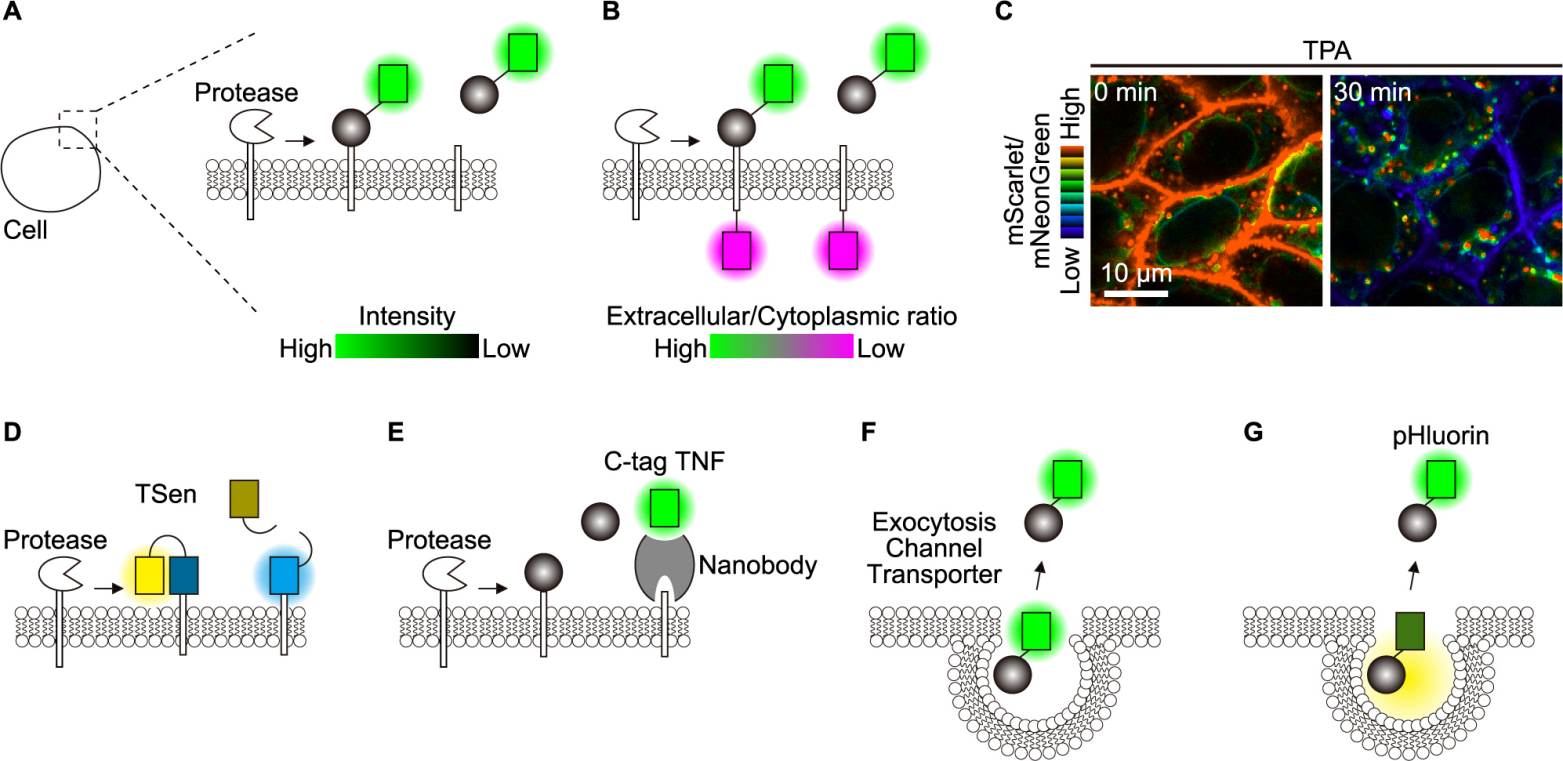
Advanced live imaging techniques for analyzing paracrine factor secretion (A) Membrane fluorescence depletion: Detection of transmembrane paracrine factor shedding through decreased cell membrane fluorescence. (B) Ratiometric fluorescence analysis: Quantification of transmembrane paracrine factor shedding via changes in the extracellular-to-cytoplasmic fluorescence ratios at the cell membrane. (C) Representative mScarlet/mNeonGreen ratio images of MDCK cells expressing EGFR ligand fused with extracellular mScarlet and cytoplasmic mNeonGreen, upon ADAM17 stimulation with 12-O-tetradecanoylphorbol 13-acetate (TPA). (D) FRET-based protease activity assay: Visualization of protease-mediated cleavage of a substrate using a FRET probe, indicated by a decrease in the YFP/CFP ratio. (E) Post-cleavage nanobody binding: Detection of cleaved TNFα using anti-C-tag nanobody (C-tag TNF) that binds to the exposed cytoplasmic domain after ADAM17-mediated cleavage. (F) Direct fluorescent labeling: Tracking of extracellular secretion using paracrine factors tagged with fluorescent proteins (FPs) or dyes. (G) pH-sensitive fluorescent reporters: Monitoring of extracellular secretion utilizing pH-sensitive FPs to detect environmental pH changes.

Many peptide ligands are expressed as transmembrane proteins or glycosylphosphatidylinositol (GPI)-anchored proteins on the plasma membrane and released by ectodomain shedding (Lichtenthaler *et al.*, 2018; Martín-de-Saavedra *et al.*, 2022). For example, ligands for epidermal growth factor receptors (EGFR ligands) are expressed as type I transmembrane proteins and released upon cleavage by ADAM-family proteases (Blobel, 2005; Zunke and Rose-John, 2017). By fusing FPs to the extracellular domain or both the extracellular and intracellular domains of the EGFR ligands, ectodomain shedding is visualized by means of the decrease in extracellular fluorescence intensity (Fig. 2A) (Bunker *et al.*, 2021) or more quantitatively by the ratio between the extracellular and intracellular FPs (Deguchi *et al.*, 2024; Inoue *et al.*, 2013; Kamezaki *et al.*, 2016) (Fig. 2B, C).

Since cell adhesion molecules may function as paracrine signaling molecules, we should include some examples belonging to this class of proteins. The postsynaptic adhesion molecule Neuroligin-1 (NLGN1), a ligand for presynaptic neurexin, is shown to be cleaved upon neuronal activation, as visualized by the extracellular fusion of an FP (Peixoto *et al.*, 2012). Similarly, thrombin-mediated shedding of CD155/PVR/Necl5, a ligand for the activating receptor DNAM-1 expressed on NK cells, is visualized by tagging FPs to both the extracellular and intracellular domains (Ichise *et al.*, 2022).

Because sheddases, enzymes responsible for the cleavage of the membrane-bound ligands, determine the timing of release, visualization of sheddase activation can be used as a surrogate marker of paracrine factor release. A good example is TSen, a biosensor designed to detect a disintegrin and metalloprotease 17 (ADAM17) activation (Chapnick *et al.*, 2015). TSen comprises an ADAM17 substrate peptide, sandwiched between fluorescent donor and acceptor for Förster resonance energy transfer (FRET). Activation of ADAM17 and resulting substrate cleavage causes a decrease in FRET, thereby visualizing ADAM17 activation (Fig. 2D). An interesting method to detect ligand shedding is reported for tumor necrosis factor (TNF) (Pinci *et al.*, 2020). C-tag TNF is a nanobody that binds to the cryptic peptide in the transmembrane domain of TNF, which is exposed only after TNF cleavage by ADAM17. By using C-tag TNF fused to an FP, live imaging of TNF shedding is achieved (Fig. 2E).

Another major pathway to release paracrine peptide ligands involves exocytosis via secretory granules. We here present insulin as an example. Although it may primarily serve as an endocrine rather than paracrine factor, extensive research on the release of insulin from β cells provides insight into the visualization of paracrine factor release (reviewed in (Gaus *et al.*, 2022)). Insulin can be directly labeled with FPs (Hatlapatka *et al.*, 2011; Michael *et al.*, 2004; Ohara-Imaizumi *et al.*, 2002, 2007; Watkins *et al.*, 2002) or indirectly with fluorescent dyes via SNAP-tag (Ivanova *et al.*, 2013) (Fig. 2F). Based on the nature of insulin maturation, a ratiometric reporter sensor, utilizing proinsulin superfolder green fluorescent protein (sfGFP) and mCherry fusions, was developed for monitoring insulin secretion (Schifferer *et al.*, 2017). Due to the small number and consequently dim fluorescence, total internal reflection fluorescence (TIRF) microscopy is preferable for imaging, which, on the other hand, limits visualization to only the close vicinity (i.e., 100–200 nm) of the cover glass. Another challenge in imaging exocytosis release arises from the low pH in secretory vesicles. Enhanced GFP (EGFP) fluorescence can be quenched in acidic conditions; therefore, interpretation of the images requires special caution (Michael *et al.*, 2004). On the other hand, this pH sensitivity of FPs can be beneficial for monitoring the secretion of paracrine factors. For example, neuropeptide Y, which is located in the same secretory granules as insulin, is fused to the pH-sensitive FP pHluorin. This neuropeptide Y-pHluorin is less fluorescent inside the acidic granules but fluoresces upon secretion into the extracellular space, allowing for the visualization of insulin release (Makhmutova *et al.*, 2017) (Fig. 2G).

The other pathways to release paracrine factors include secretion through channel opening and exosomes. Due to the difficulties in labeling small molecules that go through channels, and their small quantities and consequently faint fluorescence signals within exosomes, direct observation of the release of these paracrine factors is still difficult under the microscope. Thus, the release of these paracrine factors can be inferred by the activation of the intracellular signaling cascade within the source cells.

## Methods for Visualizing Diffusion of Paracrine Factors

**Fig. 3:**
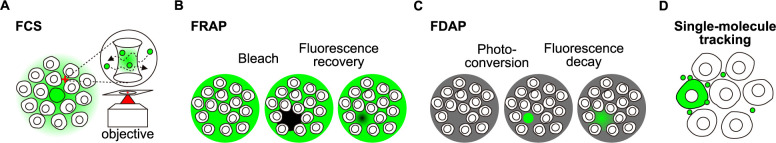
Advanced live imaging techniques for analyzing paracrine factor diffusion (A) Fluorescence correlation spectroscopy (FCS): Analysis of fluorescence fluctuations in a femtoliter-scale volume. A laser beam focused through the objective excites fluorescent molecules in a diffraction-limited, pinhole-confined volume within the tissue. FCS enables quantification of local extracellular diffusion rates of fluorescently labeled paracrine factors. (B) Fluorescence recovery after photobleaching (FRAP): Measurement of molecular mobility by photobleaching a defined area within a fluorescent field and monitoring the fluorescence recovery kinetics. FRAP allows the characterization of extracellular diffusion rates and binding-dissociation dynamics of paracrine factors tagged with FPs. (C) Fluorescence decay after photoactivation (FDAP): Spatio-temporal tracking of molecular movement using photoconvertible protein-tagged paracrine factors. Localized photoconversion and subsequent monitoring of fluorescence decay provide insights into molecular movement and clearance rates. (D) Single-molecule tracking: Direct visualization and analysis of individual paracrine factors tagged with FPs.

After leaving the source cells, paracrine factors embark on a journey to reach the target cells. This diffusion of paracrine factors has been extensively studied for morphogens, signaling molecules that form concentration gradients to provide spatial information and control cellular behaviors in a dose-dependent manner (Stapornwongkul and Vincent, 2021). Fluorescence correlation spectroscopy (FCS) is a method to visualize single-molecule dynamics of FPs. This method records fluorescence fluctuations in small volumes, which can be used as a direct measure of the molecular concentration of the protein in the tissue. Using FCS, the diffusion of fibroblast growth factor 8 (Fgf8) was visualized during zebrafish embryonic development (Fig. 3A) (Harish *et al.*, 2023; Yu *et al.*, 2009). The results suggested that not only a simple source-sink mechanism but also a ‘hindered-diffusion’ model involving the binding of Fgf8 to heparan sulfate proteoglycans (HSPGs) may explain the Fgf8 gradient in zebrafish. FCS was also applied to reveal that only a small fraction of Wnt can be freely diffusible in *Xenopus* embryos (Mii *et al.*, 2021). The Nodal/Lefty activator/inhibitor system is a key player in pattern formation during zebrafish embryogenesis (Müller *et al.*, 2013). The use of fluorescent or bioluminescent protein-tagged morphogens, including Nodal and Lefty, enabled the visualization of morphogen gradients, which are created by the balance between diffusion from source cells and clearance by target cells (Müller *et al.*, 2012; Sekine *et al.*, 2018). Furthermore, fluorescence recovery after photobleaching (FRAP) and photoconversion of FPs were used to quantitatively measure the rates of diffusion and clearance, respectively (Fig. 3B, C) (Kicheva *et al.*, 2007, 2012; Müller *et al.*, 2012; Mii *et al.*, 2021). More recently, the diffusion of Nodal and Lefty was directly observed by single-molecule tracking in combination with reflected light-sheet microscopy (Kuhn *et al.*, 2022) (Fig. 3D). The photoconversion method was also used to reveal the mechanism of gradient scaling of Decapentaplegic (Dpp), a morphogen involved in the development of *Drosophila melanogaster* (Romanova-Michaelides *et al.*, 2022). Of note, the aforementioned methods of direct labeling of paracrine factors are, in principle, applicable for tracking. For example, live imaging of endogenous or exogenous FP-tagged Wnt (Mattes *et al.*, 2018; Mii *et al.*, 2017; Pani and Goldstein, 2018) and Nodal (Liu *et al.*, 2022) showed the mode of distribution in tissue. Similarly, glutamine tagged with a fluorophore provides direct visual evidence of an activity-dependent glutamine supply from astroglial networks to presynaptic structures in live cells (Cheung *et al.*, 2022). Moreover, diffusion of FP-tagged EGFR ligands has been visualized recently (Deguchi *et al.*, 2024). This method can be enhanced by pulse-chase experiments using photoconvertible proteins, enabling precise temporal control and distinction between newly secreted and pre-existing molecules.

## Detection of the Paracrine Factor Arrival at the Target Cells

**Fig. 4:**
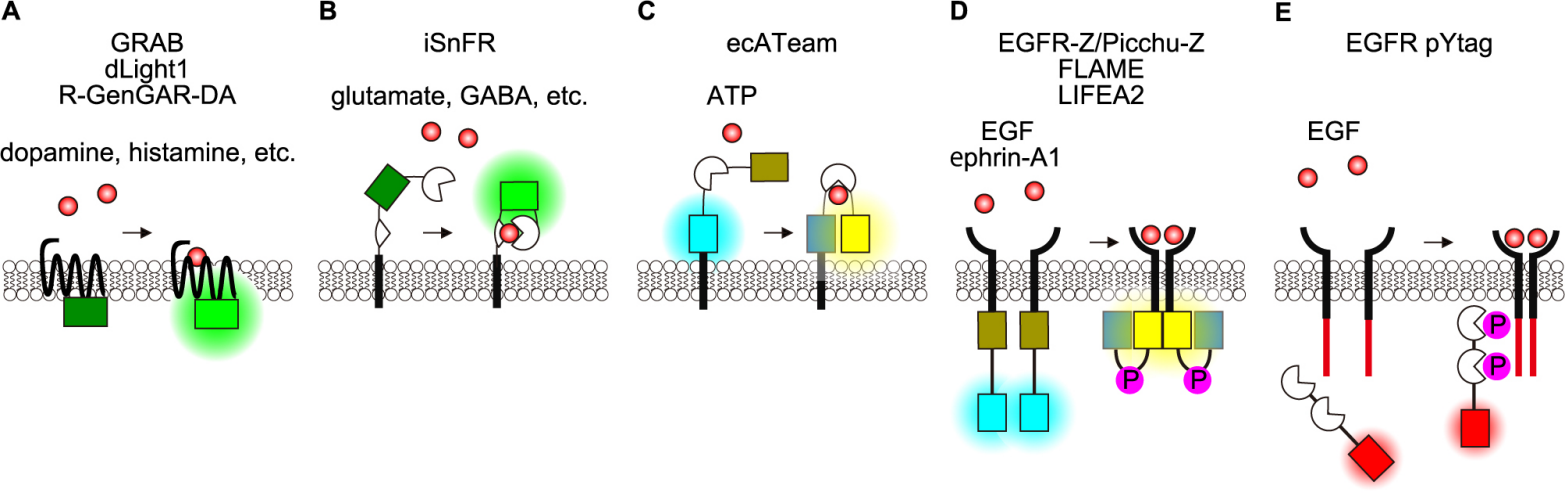
Cutting-edge receptor-based biosensors for visualizing paracrine signaling (A) GPCR-based fluorescent sensors: Detection of paracrine factor binding through an increased fluorescence of a circularly permuted FP (cpFP) fused to the intracellular surface of GPCR. (B) Intensity-based ligand-sensing fluorescent reporters (iSnFR): Extracellular paracrine factor detection via fluorescence intensity changes of a cpFP inserted into bacterial periplasmic binding proteins (PBPs). Extracellular membrane targeting is achieved by fusion with a PDGFR peptide segment. (C) Extracellular FRET sensors: Measurement of paracrine factor concentrations in the extracellular space using FRET between two FPs attached to an exposed ligand-binding domain. (D) Receptor tyrosine kinase (RTK)-based sensors: Direct fusion of phosphotyrosine-binding domains and FRET pair (CFP and YFP) to the cytoplasmic C-terminus of the RTKs. Paracrine factor binding induces RTK dimerization and phosphorylation, which is detected by changes in FRET efficiency. (E) Phosphotyrosine-tag (pYtag) system: Detection of EGF binding through recruitment of SH2 domain-fused FPs to the cytoplasmic phosphotyrosine-containing peptide of an engineered EGFR, resulting in increased membrane fluorescence.

Upon arrival at the target cells, paracrine factors bind to and activate receptors, which are in most cases located on the plasma membrane. Thus, the effective range of paracrine factors can be visualized by their binding to or activation of their cognate receptors. Cell surface receptors comprise G protein-coupled receptors (GPCRs), receptor tyrosine kinases (RTKs), cytokine receptors, ligand-gated ion channels, integrins, etc. Among them, the largest group is GPCRs, the activation of which is visualized by a number of probes including GPCR-activation-based (GRAB) sensors developed by Yulong Li’s research team (Wu *et al.*, 2022a; Zhao *et al.*, 2024) (Fig. 4A). GRAB sensors consist of a GPCR of interest and a circularly permuted FP (cpFP) fused between the transmembrane domains 5 and 6 on the intracellular surface of the GPCR. Ligand binding causes a conformational change of the cpFP, thereby increasing its fluorescence. The GRAB family sensors monitor a change in extracellular concentration of neurotransmitters including dopamine (Sun *et al.*, 2018), norepinephrine (Feng *et al.*, 2019), acetylcholine (Jing *et al.*, 2020), histamine (Dong *et al.*, 2023), ATP (Wu *et al.*, 2022b), oxytocin (Qian *et al.*, 2023), and others (Wang *et al.*, 2023). Probes similar to GRAB family sensors include dopamine sensors dLight1 (Patriarchi *et al.*, 2018, 2020) and R-GenGAR-DA1.1 (Nakamoto *et al.*, 2021), orexin sensor OxLight1 (Duffet *et al.*, 2022), GLP-1 sensor GLPLight1 (Duffet *et al.*, 2023), and norepinephrine sensors nLightG and nLightR (Kagiampaki *et al.*, 2023).

In addition to the endogenous GPCRs, bacterial periplasmic binding proteins (PBPs), components of ABC transporter complexes, are also fused to cpFPs to develop a glutamate sensor iGluSnFR (Aggarwal *et al.*, 2023; Marvin *et al.*, 2013, 2018) and derivative sensors for GABA (Marvin *et al.*, 2019), ATP (Lobas *et al.*, 2019), and serotonin (Unger *et al.*, 2020) (Fig. 4B). Note that in these probe designs, all components are expressed extracellularly.

PBPs were also applied to develop biosensors based on the principle of FRET. A glutamate sensor FLIPE comprises a truncated glutamate/aspartate binding sequence of *Escherichia coli* ybeJ/gltI, a FRET donor cyan fluorescent protein (CFP), and a FRET acceptor yellow fluorescent protein (YFP) (Okumoto *et al.*, 2005). A similar approach has been taken to develop a probe for extracellular ATP levels. The prototype ATP sensor, ATeam, comprises the ATP-binding subunit of the *Bacillus subtilis* FoF1-ATP synthase, CFP, and YFP, and monitors the cytoplasmic ATP level (Imamura *et al.*, 2009). A derivative of ATeam, ecATeam3.10, is expressed on the extracellular surface for the quantification of extracellular ATP, which, in addition to its function as an energy source, serves as an alarmin (Conley *et al.*, 2017) (Fig. 4C).

The second largest group of paracrine factor receptors is associated with cytoplasmic tyrosine kinase activities. The cytoplasmic domain of the receptor may contain the tyrosine kinase activity or may bind to periplasmic or cytosolic tyrosine kinases. Epidermal growth factor receptor (EGFR) is an archetype of the RTKs (Schlessinger, 2014). Upon binding to ligands, the receptor undergoes dimerization and exhibits increased tyrosine kinase activity, thereby phosphorylating the receptor itself and other substrates. By fusing the phosphotyrosine-binding domains and the FRET pair, CFP and YFP, to the cytoplasmic C-terminus of the receptors, reporters for EGFR or EphA2 have been developed (Itoh *et al.*, 2005; Offterdinger *et al.*, 2004; Sabet *et al.*, 2015) (Fig. 4D). In other probes for EGF, insulin, and platelet-derived growth factor, the FRET sensors were not directly connected to the cognate receptors. In these probe designs, the specificity for the ligands relies on the substrate specificity of the phosphorylation reaction (Pan *et al.*, 2019; Sato *et al.*, 2002; Seong *et al.*, 2017; Ting *et al.*, 2001). Therefore, in physiological contexts, the activation of receptors may need to be validated by other methods. Recently, a translocation reporter system pYtag biosensor for EGFR has been developed (Fig. 4E). In this probe, the immunoreceptor tyrosine-based activation motif (ITAM) motif of the T cell receptor is fused to the C-terminus of EGFR. Tandem SH2 domains derived from ZAP70 are fused to FP so that the specificity is increased (Farahani *et al.*, 2023).

## Association of Paracrine Factor Arrival with Target Cell Responses

A critical drawback of the biosensors for the receptors is that they may not be used to detect the effective signal transduction of the target cells. All GPCR sensors, including GRAB and dLight, cannot transmit signals to the heterotrimeric G proteins because the fused cpFPs hinder the signal transduction from GPCR to the heterotrimeric G proteins. Thus, the positive signals from these biosensors do not guarantee effective signal transduction in the target cells. This is true for iSnFR and ecATeam, which are not associated with the cognate receptors of the ligands. On the other hand, biosensors for RTKs hold the catalytic activity; however, it should be noted that the number of expressed biosensors is usually much higher than that of the endogenous receptors. Thus, RTK biosensors can be used to detect bath application of recombinant growth factors; however, to the best of our knowledge, they have never been used to detect endogenous growth factors released from neighboring cells.

**Fig. 5:**
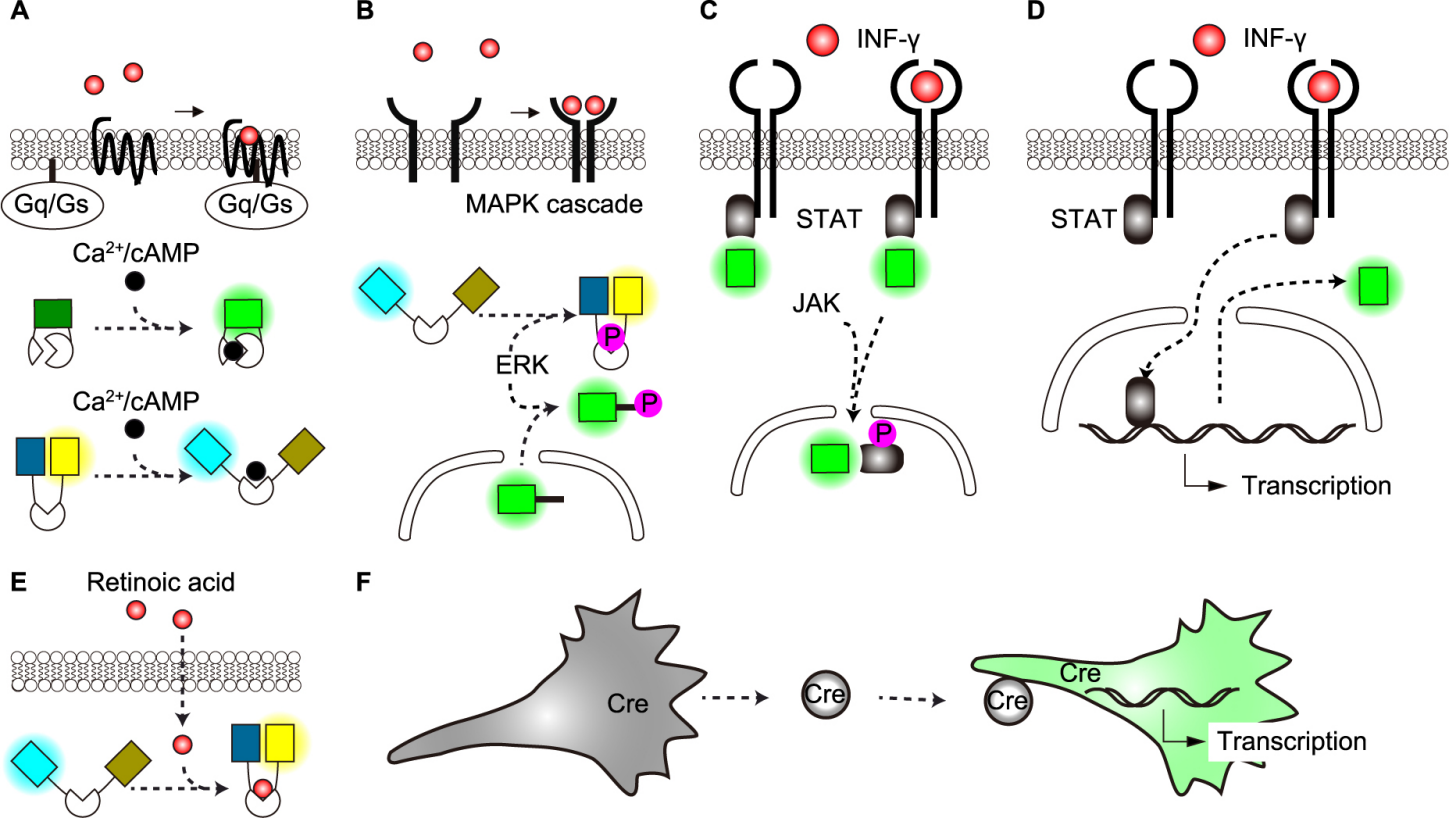
Advanced techniques for visualizing target cell responses to paracrine factors (A) Single fluorophore or FRET-based Ca^2+^/cAMP sensors: Detection of cytoplasmic Ca^2+^ or cAMP concentration increases upon GPCR activation through changes in fluorescence using cpFPs or FRET efficiency. (B) ERK activation reporters: Monitoring of extracellular signal-regulated kinase (ERK), a key downstream effector of RTKs, using either FRET-based biosensors or a kinase translocation reporter (KTR). (C) JAK/STAT pathway visualization: Real-time tracking of JAK/STAT pathway activation through observation of FP-tagged STAT protein translocation. (D) JAK/STAT pathway activation reporter: Detection of JAK/STAT pathway activation through expression of FPs by STAT-dependent transcription. (E) Cell-permeable paracrine sensors: Visualization of cell-permeable paracrine factors such as retinoic acid using FRET sensors that detect paracrine factor incorporation into target cells. (F) Extracellular vesicle (EV)-mediated signaling reporter: Visualization of EV-based paracrine signaling using a Cre recombinase-mediated FP expression system in target cells, enabling detection of successful EV delivery and content release.

The intracellular signal transduction cascades are equipped with a signal amplification mechanism. Thus, the detection of cytoplasmic second messengers or signaling molecules is much more sensitive than the detection of receptor activation and is actually used to monitor the responses invoked by paracrine factors. Canonical second messengers of GPCRs are Ca^2+^ and cyclic adenosine monophosphate (cAMP), which are located downstream of Gq and Gs/Gi, respectively. Owing to the huge effort by many research teams, the sensitivity of the sensors for Ca^2+^ (Bruton *et al.*, 2020; Dong *et al.*, 2022; Nasu *et al.*, 2021; Zhang and Looger, 2024) and cAMP (Kawata *et al.*, 2022; Massengill *et al.*, 2021, 2022; Wang *et al.*, 2022; Yokoyama *et al.*, 2024) is very high, enabling highly sensitive monitoring of Gq- or Gs-coupled GPCR activation (Fig. 5A) . For example, mechanical stress drives astrocytes to release ATP, which activates neighboring astrocytes via a Gq-coupled receptor. This ATP-mediated propagation of activation was visualized as calcium waves among the surrounding cells (Fujii *et al.*, 2017; Guthrie *et al.*, 1999; MacDonald *et al.*, 2008). Similarly, the release of prostaglandin E_2_ (PGE_2_) from a single cell has been visualized by the activation of protein kinase A (PKA), which is mediated by a Gs-coupled EP2 receptor (Watabe *et al.*, 2023).

Activation of tyrosine kinases associated directly or indirectly with the cell surface receptors triggers the MAP kinase cascade, which culminates in the activation of extracellular signal-regulated kinase (ERK). Activation of ERK can be monitored by either FRET biosensors or translocation reporters (Fig. 5B) (Harvey *et al.*, 2008; Regot *et al.*, 2014). EGFR contributes to the coordinated collective migration of epithelial cells. The diffusion of EGFR ligands is indirectly visualized as repetitive waves of ERK activation in tissue culture cells and mouse skin (Aoki *et al.*, 2017; Hiratsuka *et al.*, 2015). Cytokines such as interferon-γ constitute a large group of paracrine/endocrine factors (Altan-Bonnet and Mukherjee, 2019; Hoekstra *et al.*, 2021; Kizilirmak *et al.*, 2022). The receptors are associated with the JAK/STAT pathway. The nuclear translocation of STAT tagged with FPs (Fig. 5C) (Sanderson *et al.*, 2012; Thibaut *et al.*, 2020) or expression of FPs from STAT-dependent transcription (Fig. 5D) (Hoekstra *et al.*, 2020; Tanaka *et al.*, 2021) is used to monitor the arrival of cytokine signals. The nuclear translocation of transcription regulators is also used to monitor the activation of TNF-α receptors (Bagnall *et al.*, 2018). Meanwhile, pathway-specific transcription is used to monitor morphogens such as Hedgehog (Li *et al.*, 2018), Wnt (Akieda *et al.*, 2019; McNamara *et al.*, 2024), bone morphogenetic protein (BMP) (Zhu *et al.*, 2023), and artificial morphogens (Mizuno *et al.*, 2024; Toda *et al.*, 2020). Retinoic acid also serves as a morphogen. A stark difference from the aforementioned morphogens is that this lipophilic paracrine factor can go through the plasma membrane. Taking advantage of this nature, a FRET-based biosensor for retinoic acid has been developed to monitor the gradient of retinoic acid concentration during zebrafish development (Shimozono *et al.*, 2013) (Fig. 5E).

Extracellular vesicles (EVs), which shape metastatic niches and modify immune reactions, may be included in paracrine factors in a broad definition (Lucotti *et al.*, 2022; Nishida-Aoki and Ochiya, 2024; Pelissier Vatter *et al.*, 2021). Because EVs contain various molecules, methodologies focusing on a single molecule of interest may not be sufficient (Verweij *et al.*, 2021). An interesting idea to visualize EV-mediated intercellular communication has been published (Zomer *et al.*, 2015) (Fig. 5F). Here, the EV-secreting cells express Cre recombinase. The Cre recombinase delivered to the target cells via EVs induces the expression of FPs, thereby enabling visualization of EV arrival. Importantly, in principle, this method tells the history of EV reception and will not indicate the actual timing of the signal transduction.

## Future Directions and Emerging Technologies in Paracrine Signaling Research

**Fig. 6:**
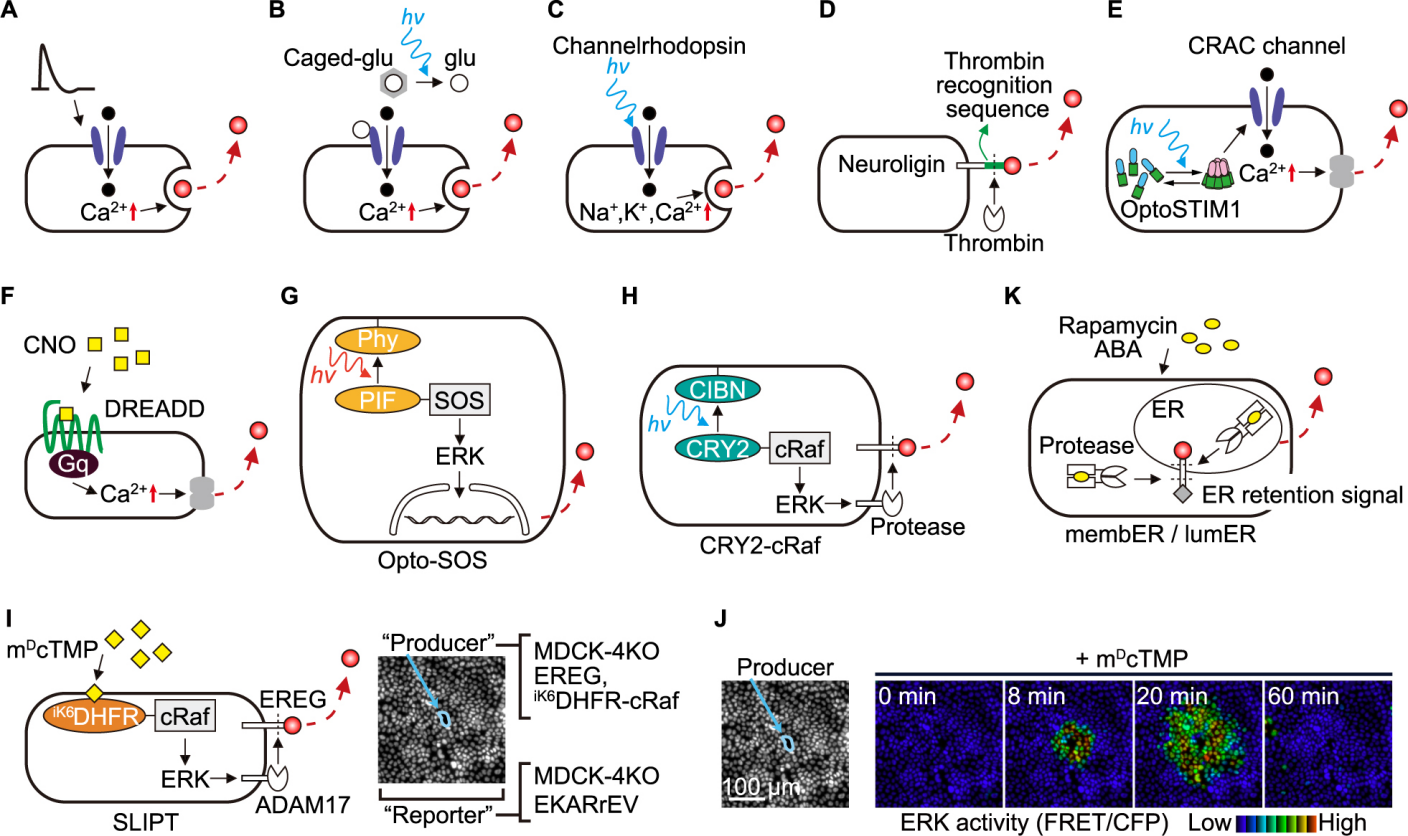
Advanced methods for inducing paracrine factor secretion (A) Neuronal depolarization: Ca^2+^-dependent neurotransmitter release at synapses triggered by neuronal depolarization. (B) Photo-uncaging of glutamate: Light-induced release of caged glutamate, stimulating Ca^2+^-dependent neurotransmitter secretion at the synapses. (C) Optogenetic neuronal activation: Light-activated channelrhodopsin-mediated cation influx, leading to neuronal activation and neurotransmitter release. (D) Thrombin-cleavable neuroligins (NLGNs): The stalk domain of NLGN1 or NLGN2 is replaced with a thrombin proteolytic recognition sequence, enabling acute and selective cleavage upon thrombin addition. (E) Chemogenetic opening of Ca^2+^ channel: Light-activated OptoSTIM1 opens CRAC channels, triggering selective Ca^2+^ influx and subsequent phospholipase A2 (PLA2) activation, resulting in PGE_2_ secretion. (F) DREADD-mediated Gq signaling: Clozapine-N-oxide (CNO) activates an artificial Gq-coupled GPCR (DREADD hM_3_), leading to mobilization of intracellular Ca^2+^ and subsequent PLA2-mediated PGE_2_ secretion. (G) Optogenetic release of IL-6-family ligands: Red-light induced membrane recruitment of phytochrome-interacting factor (PIF)-tagged SOS, a guanine nucleotide exchange factor for Ras, activating ERK and induction of IL-6-family ligand(s). (H) Optogenetic shedding of EGFR ligands: Blue light-induced membrane recruitment of cryptochrome 2 (CRY2)-fused cRaf protein, activating ERK and subsequent ADAM17-mediated shedding of EGFR. (I) (Left) Chemogenetic shedding of EGFR ligands (SLL-induced protein translocation (SLIPT) system): Synthetic myristoyl-d-Cys-tethered trimethoprim (m^D^cTMP)-induced recruitment of engineered *Escherichia coli* dihydrofolate reductase (^iK6^DHFR)-fused cRaf to the membrane, activating ERK and subsequent ADAM17-mediated shedding of EREG, an EGFR ligand. (Right) MDCK cells deficient in endogenous EGFR ligands (MDCK-4KO) expressing EREG and ^iK6^DHFR-cRaf served as source cells, while MDCK-4KO cells expressing ERK FRET biosensor (EKARrEV-NLS) were employed as reporter cells. Blue circled cells are source cells, surrounded by reporter cells. (J) ERK activity images post 10 μM m^D^cTMP treatment. (K) Chemogenetic liberation of endoplasmic reticulum (ER)-trapped paracrine factors: Chemogenetic heterodimer-mediated protease activation, resulting in the liberation of ER-trapped paracrine factors.

For long periods of time, the effect of paracrine factors has been mostly validated either by bath application of the ligands onto tissue culture cells or by observing the effects of gene knockout and/or overexpression of wild-type or mutant genes. To recapitulate the physiological responses induced by paracrine factors and to associate their release with biological outcomes, techniques are needed to trigger the secretion of paracrine factors from the cell(s) of interest. This has been well established in neuroscience, wherein depolarization of neurons drives secretion of neurotransmitters at the synapse in a Ca^2+^-dependent manner (Fig. 6A). Advancements in optogenetic tools, such as caged glutamate (Ellis-Davies, 2007) and channelrhodopsin (Boyden *et al.*, 2005), have opened the way to spatio-temporally regulate synaptic signaling at the timing of the observers’ will (Fig. 6B, C). Furthermore, the development of a system enabling acute and specific cleavage, wherein the stalk domain of NLGNs was replaced with a thrombin proteolytic recognition sequence, proved to be highly valuable in analyzing how the secretion of NLGN1 and NLGN2 in neurons impacts synaptic function (Fig. 6D) (Peixoto *et al.*, 2012; Xu *et al.*, 2024). Meanwhile, the tools for manipulating paracrine signaling in non-neuronal cells are still limited, partly due to the large number of triggering mechanisms and partly due to the lack of knowledge about the regulation thereof. Secretion of many paracrine factors from non-neuronal cells is triggered by a surge in intracellular calcium concentrations as in neuronal cells. For example, PGE_2_, a principal regulator of acute inflammation, is released into the extracellular space via Ca^2+^-dependent activation of phospholipase A2 (PLA2) (Kita *et al.*, 2019; Leslie, 2015; Park *et al.*, 2006). By using OptoSTIM1, an optogenetic tool to open plasma membrane calcium channels (Fig. 6E) (Kyung *et al.*, 2015), or DREADD hM_3_, an artificial Gq-coupled GPCR (Fig. 6F) (Rogan and Roth, 2011), activation of PLA2 and the following secretion of PGE_2_ have been visualized in epithelial cells and melanoma cells (Konishi *et al.*, 2021; Watabe *et al.*, 2024). It was found that a single discharge of PGE_2_ from the stimulated cell can activate PKA in more than 1000 neighboring epithelial cells (Watabe *et al.*, 2023).

ERK MAPK activation often leads to paracrine factor secretion. An optogenetic tool, opto-SOS, was used to activate Ras-ERK cascade and consequently to release interleukin-6 (IL-6)-family ligand(s) from NIH 3T3 cells (Fig. 6G) (Toettcher *et al.*, 2013). Since it requires a 2-hour-stimulation for the activation of neighboring cells, this is probably a transcription-dependent process. Meanwhile, ADAM17, a sheddase that cleaves EGFR ligands and TNF-α, can be rapidly activated by ERK MAPK activation. Again, optogenetic tools such as opto-SOS and opto-Raf have been developed to activate ERK and thereby trigger the secretion of EGFR ligands (Fig. 6H) (Aoki *et al.*, 2013; Kinjo *et al.*, 2019). Recently, a chemogenetic tool to activate ERK has also been developed (Fig. 6I) (Suzuki *et al.*, 2022). This new tool enabled us to visualize the liberation of EGFR ligands and resulting activation of neighboring cells in epithelial cells (Fig. 6J) (Deguchi *et al.*, 2024).

Of note, in the field of juxtacrine signaling, synthetic approaches have been adopted successfully (Bechtel *et al.*, 2021; Malaguti *et al.*, 2024). Many techniques in this research field can also be applicable to visualizing paracrine signaling. For example, synthetic regulatory secretion systems from the endoplasmic reticulum (ER) to the plasma membrane have been reported (Fig. 6K) (Praznik *et al.*, 2022; Vlahos *et al.*, 2022). In these systems, using a light-inducible or ligand-inducible heterodimerization system, viral proteases are activated to trigger the release of ER-trapped paracrine factors via exocytosis.

By combining such new tools to trigger the release of paracrine factors with the methodologies we have reviewed, we will understand how a single cell affects its neighbors and, thereby, determine the behavior of tissues, organs, and organisms.

## Author Contributions

Conceptualization: E.D., M.M., K.T.; Writing – Original Draft: E.D.; Writing – Review & Editing: E.D., M.M., K.T. Funding Acquisition: E.D., M.M., and K.T.

## Conflict of Interest

The authors declare no competing financial interests.
